# ESD-YOLO: A Method for Small-Target Termite Detection Under Complex Backgrounds

**DOI:** 10.3390/insects17080874

**Published:** 2026-08-21

**Authors:** Weiling Lu, Yuting Meng, Shan Wu, Hangjun Wang

**Affiliations:** 1 College of Engineering Technology, Jiyang College of Zhejiang A&F University, Zhuji 311800, China; 2 School of Electronic Information Engineering, Huzhou College, Huzhou 313000, China

**Keywords:** termite detection, object detection, YOLO11n, small-object detection, complex backgrounds, deep learning

## Abstract

Timely and accurate termite detection is essential for effective termite control. To address the challenges posed by the small size of termite individuals and the susceptibility of target features to background texture interference under complex backgrounds, this study proposes ESD-YOLO, a fine-grained feature-enhanced object detection model. Using YOLO11n as the baseline, ESD-YOLO redesigns the feature extraction, deep feature aggregation, and multi-scale feature fusion stages to improve the representation of small-scale termite targets under complex backgrounds. Specifically, the Efficient Multi-scale Attention (EMA) mechanism is incorporated into the C3k2 module to enhance feature discriminability between termite individuals and the background. A Spatial Pyramid Pooling-Fast with Dual Global Pooling (SPPF-DGP) module is employed to supplement deep features with global contextual information and salient response information. In addition, the DySample dynamic upsampling module is introduced to improve spatial alignment during multi-scale feature fusion and enhance boundary representation for small targets. Experimental results show that ESD-YOLO achieves Precision, Recall, mAP@0.5, and mAP@0.5:0.95 values of 95.39%, 96.33%, 97.85%, and 65.92%, respectively, with 2.67 M parameters and 6.68 G FLOPs. Compared with Faster R-CNN, RetinaNet, RT-DETR, and several YOLO-series models, ESD-YOLO demonstrates strong small-target detection and localization performance under the controlled complex-background conditions established in this study, providing a methodological reference for automated termite detection in practical settings.

## Simple Summary

Termites can damage crops, forests, buildings, and wooden structures, so timely detection is important for termite monitoring and control. However, termites are usually small, move in groups, and often appear in complex environments with soil, wood chips, stones, and termite galleries, making them difficult to detect accurately from images. In this study, we collected images of five pest termite species under complex background conditions and developed an improved computer vision model for termite detection. The model was designed to better distinguish termites from confusing background textures and to locate small termite individuals more accurately. Experimental results showed that the proposed model achieved high detection performance, with a detection accuracy of 97.85% under the main evaluation criterion and improved localization performance compared with the original model. This study provides a useful technical reference for intelligent termite monitoring and may help improve the efficiency of termite identification, population assessment, and targeted control in practical applications.

## 1. Introduction

Termites are an ancient group of eusocial insects with high species diversity; approximately 13 families, 282 genera, and nearly 3000 extant termite species have been reported worldwide [1,2]. Termites efficiently degrade lignocellulose and promote material cycling and energy flow [3], thereby playing important ecological roles, particularly in tropical and subtropical ecosystems. However, some termite species are major agricultural pests and can cause direct or indirect damage to crops such as maize, sugarcane, cassava, cotton, and peanut [4]. Termites feed on crop roots, stem bases, and woody tissues, resulting in stem boring, lodging, or plant death, which can further affect crop growth and yield [5,6]. Taking maize as an example, studies in some African production regions have shown that termite-induced yield losses typically range from 11% to 20% and can reach 50% to 100% under severe infestation [5]. Different termite groups differ in morphological characteristics, ecological habits, and damage patterns [7,8,9]; therefore, control strategies and monitoring priorities should be tailored to specific groups [10,11]. For example, subterranean termites of the genus Coptotermes respond well to baiting systems, allowing colony control through bait application; in contrast, mound-building termites show relatively weak responses to baits, and their control usually requires integrated measures such as liquid insecticide treatment, mound removal, or localized bait placement. Therefore, timely detection of termite targets and accurate identification of termite species are crucial for developing targeted monitoring, prevention, and control strategies.

Termites are polymorphic insects, and adults are difficult to collect and often exhibit complex morphological characteristics. Therefore, traditional termite identification mainly relies on morphological identification based on soldiers or winged adults, as well as molecular identification using molecular markers [12]. However, morphological identification depends heavily on expert experience, is prone to subjective errors, and is relatively inefficient, time-consuming, and labor-intensive [13,14]. Molecular identification, in turn, is limited by complex procedures and expensive equipment [15,16].

In recent years, computer vision has gradually been applied to termite identification, detection, and intelligent monitoring. For the identification of damage signs caused by subterranean termites, Jiang et al. proposed MCD-YOLOv8 to automatically detect termite-damaged areas [17]. Huang et al. used deep convolutional neural networks for termite image recognition under a single background, demonstrating the feasibility of deep learning for termite species classification [18]. For termite swarming detection and counting, Wang et al. proposed YOLOv11-ST, providing a methodological reference for early warning of agricultural and forestry disasters [19]. Zou et al. proposed YOLO-HERA, which introduces HiLo and ECPCA attention mechanisms into YOLOv8s for real-time detection of a single termite species [20]. Overall, existing termite vision studies have made preliminary progress in classification, object detection, and damage monitoring, providing useful references for intelligent termite recognition. However, the task scenarios and detection targets remain relatively scattered, and systematic research on multi-class termite individual detection under complex backgrounds is still lacking.

Compared with the limited research on termite detection, extensive studies have been conducted on the identification of other small insects and agricultural pests. Controlled image acquisition using sticky traps or insect traps has become one of the mainstream approaches in this field. For example, Rustia et al. developed a cascaded deep learning classification method for the automatic detection and identification of multiple small pests on greenhouse sticky traps [21]. Li et al. combined a spectral residual model with machine learning techniques to achieve preliminary localization of whiteflies and thrips in sticky-trap images from agricultural greenhouses [22]. In addition, Ciampi et al. developed a deep learning-based workflow for whitefly abundance estimation and spatial localization using colored sticky-trap images, providing an automated method for pest population monitoring [23]. Zhang et al. developed an automated greenhouse pest identification system based on yellow sticky traps and deep learning, enabling pest classification and counting [24]. These studies show that intelligent vision methods can support the automatic monitoring of small pest targets in relatively stable imaging systems, such as sticky traps and insect traps. However, these methods mainly focus on controlled environments or scenarios with relatively uniform backgrounds, where background interference is limited. In natural environments, complex background textures greatly increase the difficulty of extracting features from small, low-contrast targets. Therefore, methods trained on simple backgrounds still have certain limitations.

Consequently, increasing attention has been directed toward small-object pest detection under complex backgrounds. Wang et al. constructed Pest24, a large-scale agricultural pest dataset, and reported that target scale, instance number, and target clustering can affect detection performance [25]. To address challenges such as complex background interference, scale variation, and target overlap, Zhang et al. proposed AgriPest-YOLO, which improves small-pest detection through attention mechanisms, multi-scale feature aggregation, and Soft-NMS [26]. Liu et al. employed image pyramids and multi-scale feature fusion to improve the detection of tomato pests and diseases in natural environments [27]. In addition, Yolo-Pest, SP-YOLO, and YOLO-RP improve the feature representation and localization of small pest targets under complex backgrounds through strategies such as background enhancement, attention mechanisms, multi-scale feature modeling, and high-resolution feature preservation [28,29,30]. More recently, Zeng et al. addressed the query-retrieval problem in Transformer-based detectors by proposing the Heatmap-guided Embedding Learning Paradigm (HELP), which uses heatmap-guided positional embeddings to enhance foreground-relevant information and reduce the interference of background noise with target queries, providing a new perspective for improving small-object feature representation and query quality under complex backgrounds [31]. Collectively, these studies suggest that attention mechanisms, multi-scale feature modeling, and strategies that strengthen target-relevant information while reducing background interference can improve the representation and detection of small targets under complex backgrounds to some extent.

However, existing studies have mainly focused on field crop pests, tomato pests and diseases, sugar beet pests, and rice pests, many of which were imaged using automated trapping devices. Their target morphology, background characteristics, and inter-class differences differ substantially from those of termite detection tasks. In multi-class termite detection, interspecific visual differences are subtle, termite body colors can be easily confused with complex backgrounds such as wood chips, soil, stones, and termite galleries, and occlusion between adjacent individuals frequently occurs during group activity. Therefore, small-object pest detection methods designed for general complex-background scenarios may not be directly applicable to termite detection. Based on these considerations, this study develops a YOLO11n-based detection method tailored to the small target size, strong background interference, and subtle inter-class differences in termites. The model is adapted in three stages—feature extraction, deep feature aggregation, and multi-scale feature fusion—to improve multi-class termite detection and recognition under complex-background conditions. The main contributions of this study are as follows:

1. We constructed a complex-background image dataset containing five major pest termite species: *Coptotermes formosanus*, *Macrotermes barneyi*, *Odontotermes formosanus*, *Reticulitermes flaviceps*, and *Reticulitermes chinensis*.
2. We developed ESD-YOLO through a multi-stage redesign of YOLO11n tailored to multi-class termite detection under complex backgrounds. To address the small target size, strong background interference, indistinct boundary features, and subtle inter-class differences in termites, YOLO11n is redesigned at three complementary stages. C3k2-EMA is used in the feature extraction stage to strengthen target-related feature representation, SPPF-DGP is introduced in the deep feature aggregation stage to supplement global contextual and salient-response information, and DySample is employed in the multi-scale feature fusion stage to improve spatial alignment and detail recovery. Experimental results show that the resulting architecture improves the detection and localization performance of multi-class termite targets under the conditions evaluated in this study.

## 2. Materials and Methods

### 2.1. Termite Image Dataset

Currently available public termite image datasets are mainly represented by the dataset developed by Huang et al. [18], which primarily consists of segmented images of individual termites under a single controlled background and was designed mainly for termite species classification. Although image segmentation was used to obtain individual termite images with limited background content, the dataset does not provide bounding-box annotations for multi-class termite individuals under complex backgrounds and is therefore insufficient for object detection and localization in group-activity scenarios. In activity environments, termites are often mixed with background elements such as wood chips, soil, stones, and termite galleries, and may also exhibit occlusion, overlap, and blurred boundaries. To address these characteristics, we constructed an image dataset for multi-class termite individual detection under complex-background conditions.

The dataset includes five termite species collected in Zhuji City, Zhejiang Province, China: *Coptotermes formosanus*, *Macrotermes barneyi*, *Odontotermes formosanus*, *Reticulitermes flaviceps*, and *Reticulitermes chinensis*. All five species have documented distributions in Zhejiang Province and are common termite pests that cause considerable damage in China [32,33]. The samples were provided by the laboratory of the Zhuji City Termite Control Station, and their species were confirmed by professionals based on morphological characteristics to ensure the accuracy of class information. For clarity in tables and figures, abbreviations of the scientific names are used throughout this study: *Coptotermes formosanus* as CF, *Macrotermes barneyi* as MB, *Odontotermes formosanus* as OF, *Reticulitermes flaviceps* as RF, and *Reticulitermes chinensis* as RC.

**Figure 1:**
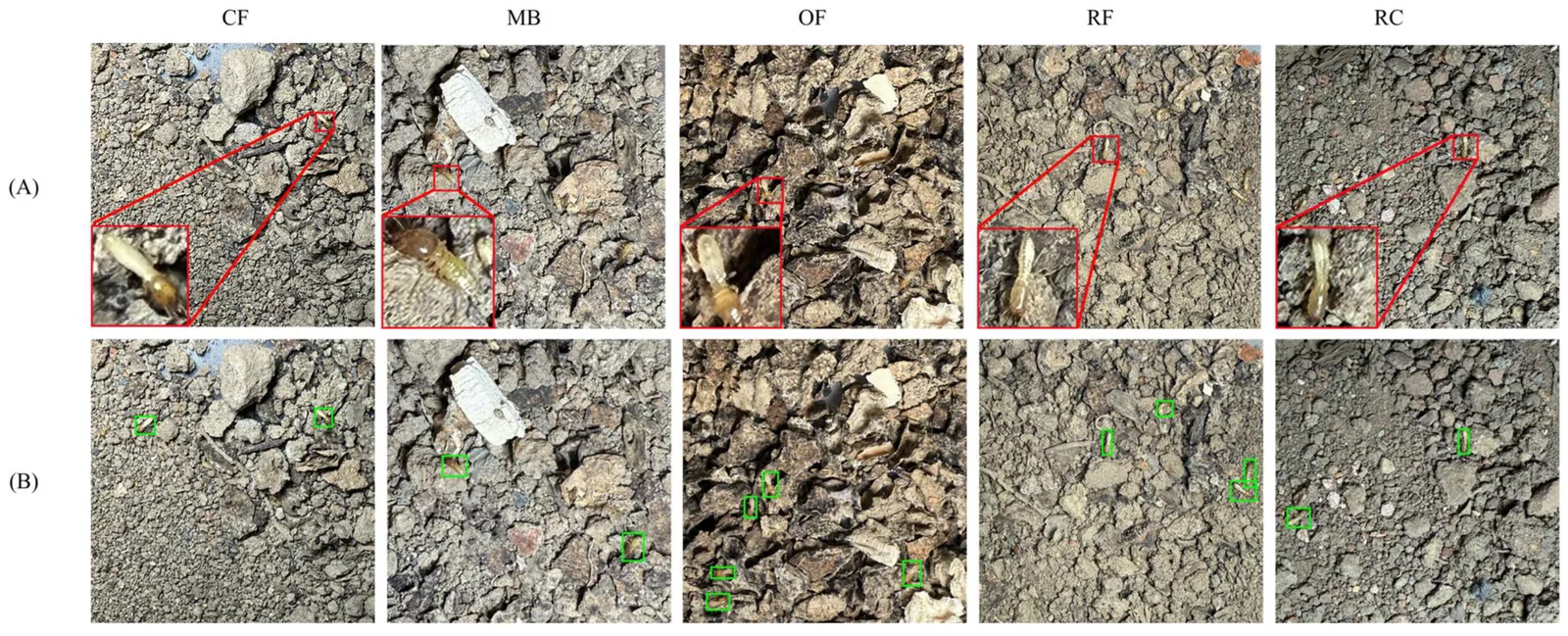
Representative images and manual annotation examples from the termite dataset. (**A**) Representative images with enlarged views of termite targets, where red boxes indicate the regions selected for enlargement; (**B**) corresponding ground-truth bounding-box annotations, shown in green.

To simulate the background conditions encountered during termite activity while ensuring that species identification and bounding-box annotations could be reliably verified, images of different termite species were collected in acrylic rearing boxes containing wood chips, soil, small stones, and termite galleries. Compared with uncontrolled field acquisition, controlled image collection allows the imaging distance, camera angle, and illumination conditions to be regulated, facilitating the acquisition of annotation samples with reliable class information and relatively clear target boundaries [34]. The image acquisition system consisted of a robotic-arm stand (CamScanner, INTSIG PTE. LTD., Singapore), a supplementary light source (CamScanner, INTSIG PTE. LTD., Singapore), and an iPhone 16 Pro Max (Apple Inc., Cupertino, CA, USA). During image acquisition, the smartphone was fixed above the rearing box for top-view imaging. Images were collected from May to June 2026, with 10 acquisition sessions conducted for each termite species, resulting in 50 sessions in total. An acquisition session was defined as one continuous, uninterrupted image-capture process, during which images were acquired at an interval of 10 s. The original image resolution was 3024 × 3024 pixels. Different physical rearing boxes were used for different acquisition sessions, with the arrangement of soil, wood chips, and other background materials and the illumination conditions varied between sessions. Within each acquisition session, the imaging device, camera angle, and illumination conditions were kept stable. To reduce temporal redundancy caused by continuous sampling, adjacent images within each acquisition session were screened for similarity, and redundant images with highly similar termite postures, target distributions, and background layouts were removed. Representative acquisition examples are shown in Figure 1A.

After image screening, the retained images were uniformly resized to 640 × 640 pixels and manually annotated with bounding boxes at this resolution using LabelImg 1.8.6. The annotations included the target class and bounding-box coordinates and were subsequently converted to YOLO format. During annotation and quality control, each individually identifiable termite was treated as a separate annotation instance. Bounding boxes were drawn as tightly as possible around the visible body and included identifiable appendages such as antennae and legs. Overlapping termites were annotated separately when individual termites could still be distinguished. Partially occluded targets were retained when their species could be reliably identified, whereas severely occluded individuals were excluded when reliable classification was not possible. Termites located at image boundaries and only partially visible were retained when sufficient identifiable features remained for reliable species determination. The dataset followed a quality-control procedure involving one primary annotator and two reviewers. All images were initially annotated by the first author and subsequently checked and finally reviewed by the second and third authors. Class labels were assigned according to the termite species identified by professionals based on morphological characteristics before image acquisition. Representative images of the five termite species and the corresponding manual annotations are shown in Figure 1B.

The final termite dataset comprised five species, with 10 acquisition sessions conducted for each species, resulting in 3160 images and 7421 valid termite instances. The dataset was divided into training, validation, and test sets at an approximate ratio of 7:1:2 using acquisition sessions as the partitioning unit. These three subsets were used for model training, model selection and hyperparameter tuning, and final performance evaluation, respectively. During training, online random data augmentation was applied only to the training set, including HSV color augmentation, random translation, scale augmentation, horizontal flipping, and Mosaic augmentation. No data augmentation was applied to the validation or test sets. The numbers of images and instances for each class and the dataset split are summarized in Table 1.

To quantitatively characterize the scale of termite targets in the detection images, the bounding-box areas of all valid termite instances in the 640 × 640-pixel images and their proportions relative to the total image area were calculated, as shown in Table 2. The median bounding-box area was 1410.01 pixels^2^, accounting for 0.34% of the total image area. The first quartile (Q1) and third quartile (Q3) were 1144.00 pixels^2^ and 1739.98 pixels^2^, corresponding to 0.28% and 0.42% of the image area, respectively. Overall, the bounding-box size distribution of termite targets was relatively concentrated, with each individual target occupying only a small proportion of the entire image.

### 2.2. ESD-YOLO Module

The YOLO series, first proposed by Redmon et al. in 2016 [35], represents a typical family of single-stage object detection algorithms. It performs object class prediction and bounding-box regression in a single forward pass and is characterized by fast detection speed, a simple architecture, and ease of deployment. Through continuous development, the YOLO series has been optimized in terms of feature extraction, feature fusion, and detection head design.

**Figure 2:**
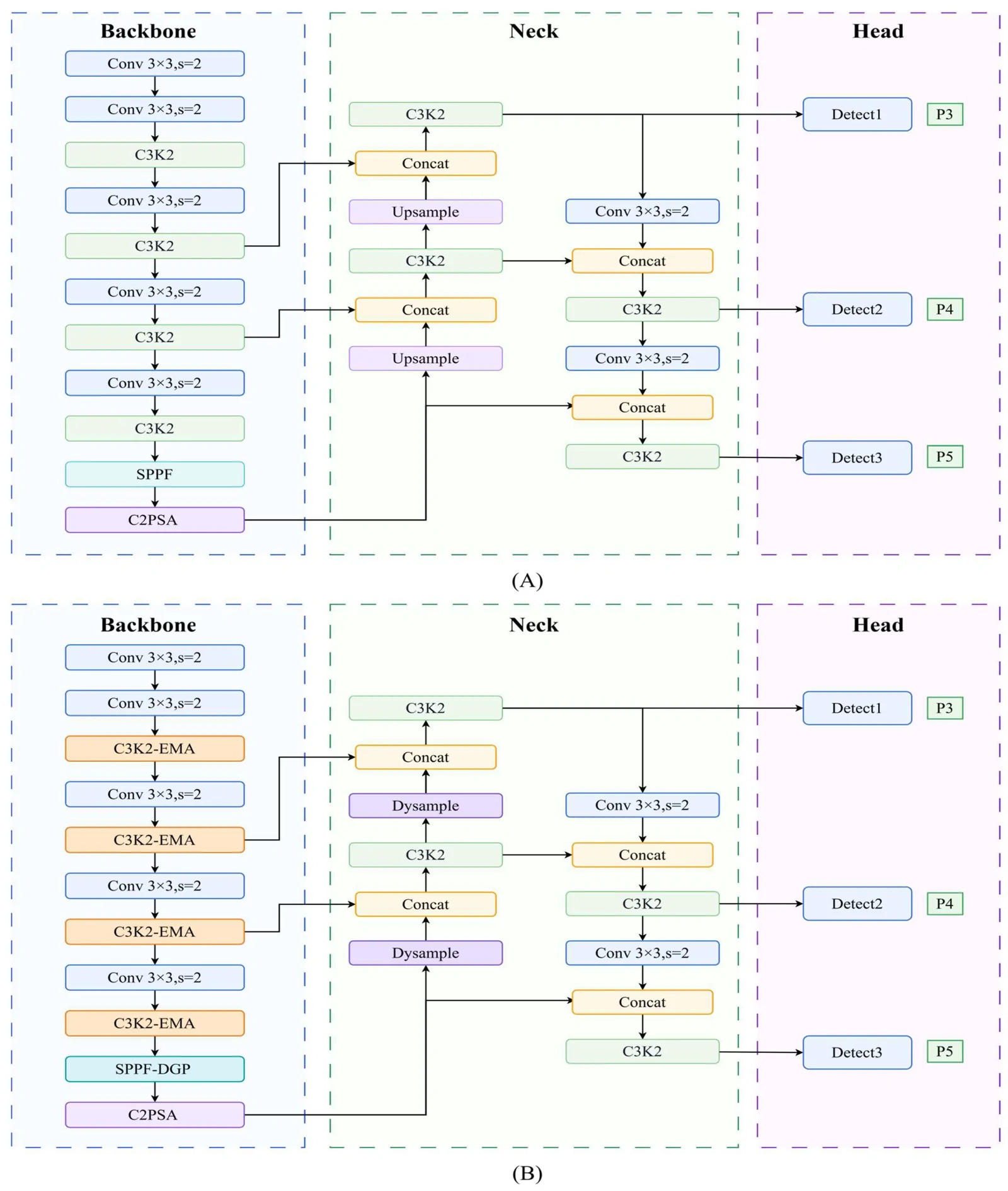
Comparison of the network architectures of YOLO11n and ESD-YOLO. (**A**) YOLO11n; (**B**) ESD-YOLO. Arrows indicate the direction of feature flow, and different colors are used to distinguish different network modules.

YOLO11 maintains the advantages of real-time detection and provides five model configurations—n, s, m, l, and x—to meet different requirements for detection accuracy and computational cost. Its network architecture is shown in Figure 2A. Considering the need for a lightweight model and low computational cost in termite detection, YOLO11n was selected as the baseline model in this study. Based on YOLO11n, ESD-YOLO was proposed, and its overall structure is shown in Figure 2B.

ESD-YOLO is improved in three aspects: feature extraction, deep feature aggregation, and multi-scale feature fusion. In the feature extraction stage, C3k2-EMA is adopted as a feature enhancement component in the backbone to improve the representation of termite-related features. In the deep feature aggregation stage, the original SPPF module is replaced with SPPF-DGP, which introduces global average pooling and global max pooling while retaining multi-scale pooled features to enhance the modeling of global contextual information and salient response regions. In the feature fusion stage, DySample dynamic upsampling is used instead of the original nearest-neighbor upsampling in the neck to improve spatial alignment and detail recovery during multi-scale feature fusion.

#### 2.2.1. C3k2-EMA Module

EMA is a lightweight multi-scale attention module that enhances feature representation while controlling computational cost [36]. Based on channel grouping, EMA draws on the directional positional encoding strategy used in Coordinate Attention (CA) [37] and recalibrates features through multi-scale parallel branches and cross-spatial interactions, thereby improving spatial feature representation with low computational overhead.

**Figure 3:**
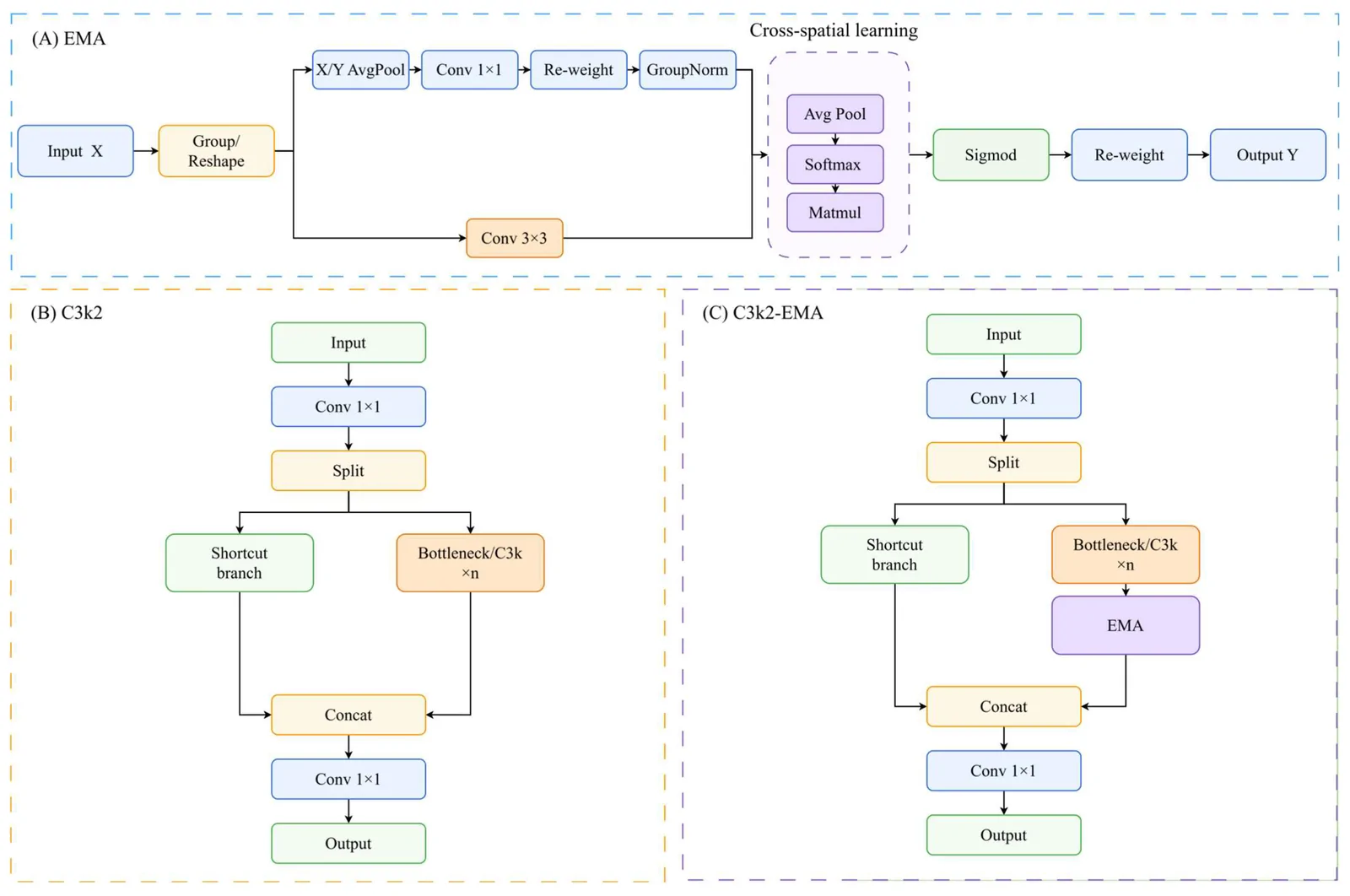
Structure of the EMA and C3k2-EMA module. (**A**) EMA module; (**B**) C3k2 module; (**C**) C3k2-EMA module. Arrows indicate the direction of feature flow, and different colors are used to distinguish different operations or modules.

As shown in Figure 3A, let the input feature be $X\in R^{B\times C\times H\times W}$, where B, C, H, and W denote the batch size, number of channels, feature map height, and feature map width, respectively. EMA first divides the input feature into G sub-feature groups along the channel dimension and merges the grouping dimension into the batch dimension for parallel computation. Subsequently, each grouped feature is fed into two parallel branches. One branch extracts directional positional information through one-dimensional global average pooling along two spatial directions and generates directional attention weights after feature concatenation and 1 × 1 convolution. The other branch uses 3 × 3 convolution to extract local neighborhood features. EMA then performs global pooling and Softmax normalization on the two branches separately, followed by matrix multiplication with the spatial features from the other branch to achieve cross-spatial interaction and generate a pixel-wise attention map. Finally, this attention map is used to reweight the grouped features, and the outputs of all groups are restored to the original channel dimension. Through this process, EMA integrates directional positional information, local neighborhood information, and cross-spatial dependencies, enhancing feature responses in termite target regions while suppressing background texture interference. The basic process can be expressed as follows:

(1) $$X_{g}={Reshape}_{G}(X),X_{g}\in R^{\left(\left(B\cdot G\right)\times \left(C/G\right)\times H\times W\right)}$$

(2) $$\left[A_{h},A_{w}\right]=SplitConv_{1\times 1}\left[Concat\left({Pool}_{H}\left(X_{g}\right),Pool_{W}\left(X_{g}\right)\right)\right]$$

(3) $$F_{1}=GN\left[X_{g}⊙\sigma \left(A_{h}\right)⊙\sigma \left(A_{w}\right)\right],F_{3}=Conv_{3\times 3}\left(X_{g}\right)$$

(4) $$M=\sigma Softmax\left[GAP\left(F_{1}\right)\right]\cdot {\bar{F}}_{3}+Softmax\left[GAP\left(F_{3}\right)\right]\cdot {\bar{F}}_{1}$$

(5) $$Y=Reshape_{G}^{\left(-1\right)}\left(X_{g}⊙M\right)$$

Here, B denotes the batch size, G denotes the number of groups, and $X_{g}$ denotes the features after channel grouping and reordering. $A_{h}$ and $A_{w}$ denote the directional attention weights in the two spatial directions, respectively. $F_{1}$ denotes the direction-enhanced features, and $F_{3}$ denotes the local features extracted by 3 × 3 convolutions, $GN\left(\cdot \right)$ denotes GroupNorm, $\sigma \left(\cdot \right)$ denotes the Sigmoid activation function, ⊙ denotes element-wise multiplication, and $GAP\left(\cdot \right)$ denotes global average pooling. M denotes the pixel-wise attention map generated through cross-spatial interaction, and $Softmax\left(\cdot \right)$ is used to normalize the channel descriptors. $Reshape_{G}^{-1}\left(\cdot \right)$ denotes the inverse grouping reshape operation that restores the grouped features to the original channel dimension, and Y is the output feature of the EMA module.

Although the C3k2 module in YOLO11n provides effective lightweight feature extraction, its selective representation of key target regions remains limited in detection scenarios involving complex backgrounds, small targets, and low contrast. Xu et al. incorporated the EMA mechanism into the C3k2 structure of YOLO11 in EM-YOLO for electronic component detection [38]. Building on this design, we further adapted the integration of EMA within C3k2 to the characteristics of termite detection, including small target scale, low contrast, and susceptibility to complex background textures. The resulting C3k2-EMA is used as a feature-enhancement component in the backbone of ESD-YOLO, as shown in Figure 3C. Termites are typically milky white or pale yellow and often exhibit low color contrast against backgrounds such as soil, wood chips, and termite galleries. In addition, wood-chip textures, soil particles, and irregular background edges can interfere with the extraction of termite contours and detailed features. Through cross-spatial attention-based feature reweighting, C3k2-EMA can enhance responses in termite-related regions while reducing the influence of irregular background textures.

#### 2.2.2. SPPF-DGP Module

**Figure 4:**
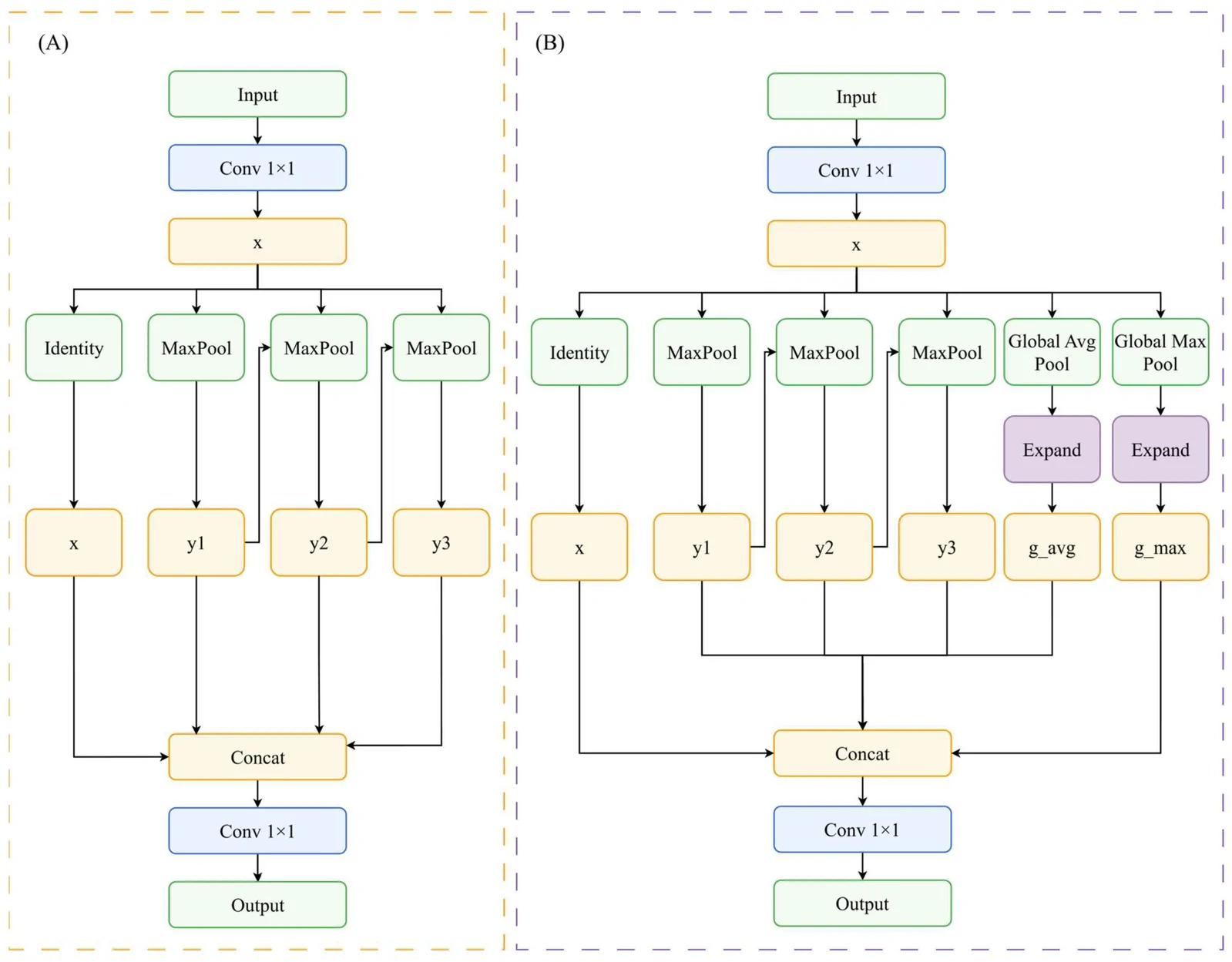
Structural comparison of the SPPF and SPPF-DGP modules. (**A**) SPPF module; (**B**) SPPF-DGP module. Arrows indicate the direction of feature flow, and different colors are used to distinguish different operations or branches.

Spatial Pyramid Pooling-Fast (SPPF) is an important feature aggregation module in the YOLO11n backbone, primarily used to expand the receptive field and fuse multi-scale contextual information. Unlike the traditional SPP architecture, which uses multiple pooling kernels of different sizes to extract features in parallel, SPPF consecutively stacks several 5 × 5 max-pooling operations to obtain feature representations similar to multi-scale pooled features with lower computational cost. Its basic process is as follows: the input features first undergo a 1 × 1 convolution for channel reduction and then sequentially pass through multiple max-pooling operations to obtain feature responses with different receptive fields. Finally, the original branch and the pooled branches are concatenated, and feature fusion is performed through a 1 × 1 convolution. The structure of SPPF is shown in Figure 4A. This module enhances the multi-scale representation of deep features while maintaining high computational efficiency and is therefore widely used in the deep feature extraction stage of YOLO-series detection networks.

However, the original SPPF mainly relies on max-pooling operations to extract salient response regions. Although this design can highlight strong local responses, its ability to model global feature distributions and contextual information is relatively limited. In termite detection, termite activity is often associated with environmental features such as termite galleries, eroded wood, soil particles, and wood chips, which can provide useful contextual cues for identifying target regions. If the model relies only on local maximum responses, it may overemphasize non-target high-response regions such as wood chips, stones, or termite-gallery edges, thereby increasing the risk of false positives. Li et al. improved SPPF in IF-YOLO by introducing adaptive average pooling and adaptive max pooling [39]. Based on the original SPPF, this study introduces global average pooling and global max pooling branches and denotes the resulting structure as SPPF-DGP, as shown in Figure 4B. Global average pooling is used to supplement information about the overall feature distribution, whereas global max pooling preserves salient responses across the feature map. By fusing multi-scale max-pooled features with dual global pooling features, SPPF-DGP can jointly exploit local salient information and global contextual information, thereby reducing the influence of non-target textures in complex backgrounds on detection results.

#### 2.2.3. DySample Module

In the Neck of YOLO11n, upsampling is used to restore deep, low-resolution features to a higher spatial scale and fuse them with shallow features. The original YOLO11n adopts nearest-neighbor upsampling, which is computationally simple but uses fixed sampling positions and therefore cannot adapt well to local feature structures. For small-object termite detection, this fixed upsampling strategy may weaken target boundaries and spatial details, thereby affecting subsequent multi-scale feature fusion.

To improve feature restoration quality, we introduce the DySample dynamic upsampling module [40] into the Neck of YOLO11n to replace the original nn.Upsample operation. DySample reformulates the upsampling process from a point-sampling perspective. Instead of directly generating dynamic convolution kernels, it predicts sampling offsets from the input features and uses the grid_sample function for feature resampling. This method avoids complex dynamic convolutions and additional CUDA operators, while requiring few parameters, incurring low computational cost, and being easy to integrate into lightweight detection networks.

**Figure 5:**
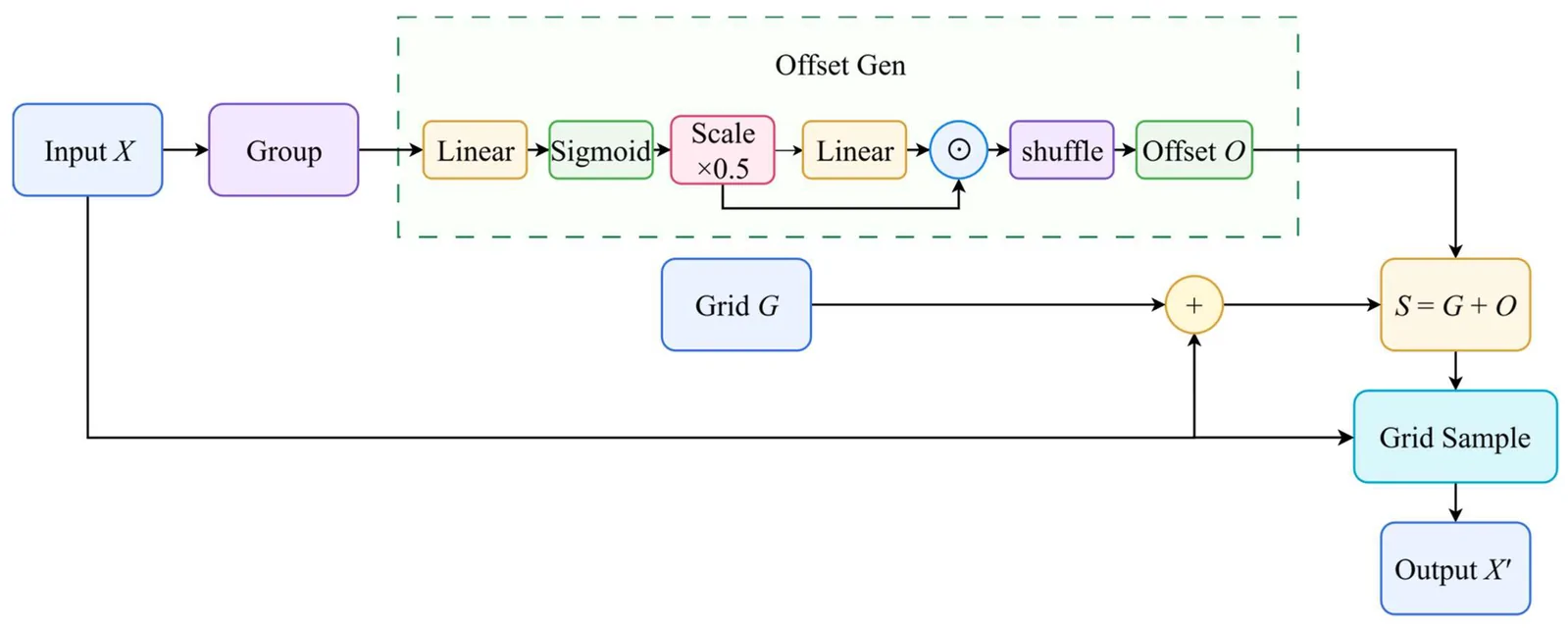
Structure of the DySample module. Arrows indicate the direction of feature flow, and different colors are used to distinguish different operations or branches.

The structure of DySample is shown in Figure 5. Let the input feature be $X\in R^{B\times C\times H\times W}$, and let the upsampling factor be s. First, the input feature X is grouped and fed into the offset generator. The offset generator consists of linear mapping, Sigmoid activation, range scaling, element-wise multiplication, and a shuffle operation, and is used to generate the sampling offset O. Next, O is added to the regular sampling grid G to obtain the dynamic sampling point set S. Finally, the input feature X is resampled according to S to obtain the upsampled output feature $X^{\prime }\in R^{B\times C\times sH\times sW}$. The basic process can be expressed as follows:

(6) $$O=0.5\sigma \left(F_{1}\left(X_{g}\right)\right)⊙F_{2}\left(X_{g}\right)$$

(7) S=G+O

(8) $$X^{\prime }=\mathrm{GridSample}\left(X,S\right),X^{\prime }\in R^{B\times C\times sH\times sW}$$

Here, $X_{g}$ represents the grouped input features; $F_{1}\left(\cdot \right)$ and $F_{2}\left(\cdot \right)$ represent the two linear mapping functions in the offset generator, respectively; $\sigma \left(\cdot \right)$ represents the Sigmoid activation function; ⊙ denotes element-wise multiplication; and + denotes element-wise addition. O, G, S, and $X^{\prime }$ denote the dynamic offset, regular sampling grid, dynamic sampling point set, and upsampled output feature, respectively. By dynamically adjusting the sampling positions, DySample enables the upsampling process to better adapt to local feature distributions, thereby improving spatial alignment in multi-scale feature fusion.

In the proposed model, DySample is used in the top-down feature fusion paths from P5 to P4 and from P4 to P3. Termites are small, and occlusion and overlap commonly occur during group movement. In addition, termite edges are often difficult to distinguish from backgrounds such as soil, wood chips, and termite galleries. Traditional nearest-neighbor upsampling uses fixed sampling positions and cannot adapt well to local boundary and texture variations, which may weaken edge information or cause spatial misalignment. In contrast, DySample adaptively adjusts sampling positions by predicting sampling offsets, helping to improve the alignment between deep semantic features and shallow spatial features. This reduces the interference of background information with target-region features and enhances the boundary representation of small termite targets.

## 3. Experimental Results and Analysis

### 3.1. Experimental Environment and Parameter Settings

The experiments in this study were conducted on a 64-bit Windows 11 Pro operating system. The hardware platform consisted of two AMD EPYC 7352 24-core processors, providing 48 physical cores and 96 logical threads in total, with a clock speed of 2.30 GHz and 64 GB of system memory. Model training was performed on an NVIDIA GeForce RTX 4090 GPU with 24 GB of GPU memory. The software environment was based on the YOLOenv environment created using Anaconda, with Python 3.11.15, PyTorch 2.11.0+cu128, CUDA 12.8, cuDNN 9.1.9, and Ultralytics 8.3.9.

During training, the input image size was set to 640 × 640, the number of training epochs was 300, the batch size was 48, and SGD was used as the optimizer. Except for the explicitly specified parameters, all other training parameters and data augmentation strategies followed the default Ultralytics configuration. The main training parameters are shown in Table 3.

### 3.2. Evaluation Criteria

To comprehensively evaluate model performance in the termite object detection task, Precision, Recall, mAP@0.5, mAP@0.5:0.95, Params, FLOPs, and FPS were selected as evaluation metrics. Precision and Recall were used to assess the reliability of classification and target detection, whereas mAP@0.5 and mAP@0.5:0.95 were used to evaluate overall detection performance under different IoU thresholds. Params and FLOPs were used to measure model size and computational complexity, respectively, while FPS was used to quantify practical inference speed. The formulas for these evaluation metrics are as follows:

(9) $$Precision=\frac{TP}{TP+FP}$$

(10) $$Recall=\frac{TP}{TP+FN}$$

(11) $$AP=\int_{0}^{1}P\left(R\right)dR$$

(12) $$\mathrm{mAP}=\frac{1}{T}\sum_{t=1}^{T}AP_{t}$$

(13) $$FPS=\frac{N}{t}$$

In Equations (9) and (10), Precision denotes detection precision, Recall denotes detection recall, and TP, FP, and FN denote the numbers of true positives, false positives, and false negatives, respectively. In Equation (11), AP denotes average precision, which is calculated as the area under the Precision–Recall curve, where P denotes precision and R denotes recall. In Equation (12), mAP denotes mean average precision, T denotes the total number of classes, and $AP\left(t\right)$ denotes the average precision of the t-th class. In Equation (13), FPS denotes the number of image frames processed per second, N denotes the total number of images evaluated, and t denotes the total time required to complete preprocessing, model inference, and postprocessing for all test images.

### 3.3. Ablation Experiment

To evaluate the contribution of each improved module to termite object detection performance, YOLO11n was used as the baseline model. Under the same dataset split, training parameters, and test-set evaluation conditions, the C3k2-EMA, DySample, and SPPF-DGP modules were introduced individually, and models with different module combinations were further constructed for ablation experiments. The results are shown in Table 4.

As shown in Table 4, the YOLO11n baseline model achieved Precision, Recall, mAP@0.5, and mAP@0.5:0.95 values of 93.32%, 97.01%, 97.50%, and 63.95%, respectively, on the test set, indicating that it already provided a strong detection baseline for the dataset used in this study. Among the single-module improvements, SPPF-DGP increased mAP@0.5:0.95 to 64.48%, suggesting that dual global pooling helps supplement contextual information in deep features. DySample increased mAP@0.5:0.95 to 64.99%, indicating that dynamic upsampling improves spatial alignment during multi-scale feature fusion. C3k2-EMA achieved Precision and mAP@0.5:0.95 values of 95.57% and 65.51%, respectively, showing the most pronounced improvement among the single-module experiments. This result indicates that the attention mechanism can enhance effective feature responses in termite target regions.

In the combined-module experiments, ESD-YOLO incorporated C3k2-EMA, DySample, and SPPF-DGP simultaneously, achieving an mAP@0.5:0.95 of 65.92%. This represents a 1.97 percentage point improvement over the baseline model and is the highest value among all ablation configurations. Meanwhile, the number of parameters increased from 2.36 M to 2.67 M, and FLOPs increased from 6.44 G to 6.68 G, indicating only a limited increase in computational cost. The results also show that different module combinations did not lead to linear improvements across all metrics. For example, some configurations achieved higher Recall or mAP@0.5 than ESD-YOLO. Nevertheless, ESD-YOLO achieved the highest mAP@0.5:0.95 while maintaining high Precision and mAP@0.5, indicating a favorable balance between detection accuracy and localization performance.

### 3.4. Comparative Experiment

To further evaluate the detection performance of ESD-YOLO, mainstream single-stage and two-stage object detection models were selected for comparative experiments. All models used the same training, validation, and test-set splits, with the input image size uniformly set to 640 × 640 pixels and the number of training epochs set to 300. Except for differences inherent to the model architectures, the remaining training strategies and parameter configurations were kept as consistent as possible to minimize the influence of experimental conditions on the comparisons. In addition to the overall performance comparison, multiple independent training runs were conducted to evaluate the stability and reproducibility of YOLO11n and ESD-YOLO under different random seeds. The detection performance of the two models was also compared across different termite classes and relative target scales. These experiments provide a comprehensive evaluation of ESD-YOLO in terms of overall detection performance, independent training stability, class-wise performance, and target-scale performance.

#### 3.4.1. Comparison of Overall Performance Metrics

To evaluate the overall detection performance of ESD-YOLO, Faster R-CNN, RetinaNet-ResNet50, RT-DETR-R18, YOLOv8n, YOLOv9t, YOLOv10n, YOLO11n, and YOLO12n were selected for comparison. The experimental results are shown in Table 5. The FPS of all models was measured on an NVIDIA GeForce RTX 4090 GPU, with the timing covering image preprocessing, model inference, and postprocessing of the detection results. Each model was evaluated three times on the complete test set, and the average FPS over the three runs is reported in the table.

As shown in Table 5, Faster R-CNN, RetinaNet-ResNet50, and RT-DETR-R18 achieved mAP@0.5:0.95 values of 49.44%, 56.89%, and 57.59%, respectively, on the dataset used in this study, which were lower than those of the lightweight YOLO models. In addition, the Params and FLOPs of these three models were substantially higher than those of ESD-YOLO. In particular, the FLOPs of Faster R-CNN and RetinaNet-ResNet50 reached 269.02 G and 257.18 G, respectively, far exceeding the 6.68 G of ESD-YOLO. These results indicate that, on the present dataset, larger model sizes did not yield corresponding accuracy gains in small-target termite detection. One possible explanation is that termite targets are small and strongly affected by background texture interference. Although heavier backbone networks and detection frameworks generally provide stronger global feature modeling capabilities, they may not necessarily preserve and exploit the local details and weak boundary information of small targets effectively, thereby limiting bounding-box localization performance.

In contrast, YOLOv8n, YOLOv9t, YOLOv10n, YOLO11n, and YOLO12n all achieved mAP@0.5 values above 97%, indicating that lightweight YOLO models are well suited to termite target detection under complex backgrounds. These models adopt a single-stage detection framework and perform joint detection of targets at different scales through multi-scale feature extraction, cross-layer feature fusion, and multi-scale prediction. With relatively low parameter counts and computational costs, they maintain strong detection performance in terms of mAP@0.5 while balancing target classification and bounding-box regression. Among these models, YOLO11n achieved the highest Recall of 97.01%, indicating strong termite detection capability. YOLO12n had the lowest FLOPs, at 5.98 G, corresponding to the lowest theoretical computational cost among the compared models. YOLOv9t achieved an mAP@0.5:0.95 of 63.69%, showing relatively strong bounding-box localization performance. In terms of practical inference speed, YOLOv8n achieved the highest FPS of 48.89, followed by YOLOv10n and YOLO11n at 45.63 and 40.99 FPS, respectively, whereas YOLOv9t and YOLO12n achieved 23.58 and 29.99 FPS. The variation in FPS was not fully consistent with the trends in Params and FLOPs, indicating that model size and theoretical computational complexity do not completely reflect practical inference speed.

ESD-YOLO achieved favorable overall detection performance among the compared models, with Precision, mAP@0.5, and mAP@0.5:0.95 values of 95.39%, 97.85%, and 65.92%, respectively, all of which were the highest in Table 5. Compared with the baseline YOLO11n, ESD-YOLO improved mAP@0.5 and mAP@0.5:0.95 by 0.35 and 1.97 percentage points, respectively. The more pronounced improvement in mAP@0.5:0.95 suggests that the proposed modifications mainly contributed to more precise bounding-box localization. This improvement may be associated with the enhancement of termite-related features by EMA, the supplementation of deep global contextual and salient response information by SPPF-DGP, and the improved spatial alignment of multi-scale features by DySample.

In terms of Recall, ESD-YOLO was 0.68 percentage points lower than YOLO11n but still maintained a high level, while its Precision increased by 2.07 percentage points, indicating that the model reduced false positives while maintaining strong target detection capability. ESD-YOLO contained 2.67 M parameters and required 6.68 G FLOPs. Although both values were slightly higher than those of YOLO11n, they remained substantially lower than those of Faster R-CNN, RetinaNet-ResNet50, and RT-DETR-R18. In terms of inference efficiency, ESD-YOLO achieved 29.81 FPS, lower than the 40.99 FPS of YOLO11n, indicating that the performance improvement was accompanied by additional inference-time overhead. Its FPS was higher than those of Faster R-CNN, RetinaNet-ResNet50, and YOLOv9t, and was comparable to those of RT-DETR-R18 and YOLO12n. Overall, ESD-YOLO improved the detection accuracy and localization performance of multi-class termite targets under complex backgrounds while maintaining relatively low model complexity, although its practical inference efficiency still has room for further optimization.

#### 3.4.2. Comparison of Independent Training Stability

To evaluate the stability and reproducibility of the performance improvements under different random training conditions, YOLO11n and ESD-YOLO were each independently trained five times using random seeds of 0, 1, 2, 3, and 4, while the other main training configurations were kept unchanged. The best-performing model obtained from each training run was evaluated on the same test set using identical evaluation settings. The results of the individual runs, together with their means and sample standard deviations, are presented in Table 6 and Table 7, respectively.

As shown in Table 6, the detection performance of both YOLO11n and ESD-YOLO showed some variation across different random seeds. Notably, ESD-YOLO achieved higher mAP@0.5:0.95 than the corresponding YOLO11n run in all five independent training runs, indicating good consistency of this performance advantage under different random training conditions. Table 7 further presents the means and sample standard deviations of the five independent training runs. ESD-YOLO achieved mean Precision, Recall, and mAP@0.5 values of 94.69%, 97.05%, and 97.85%, respectively, representing improvements of 0.41, 0.11, and 0.15 percentage points over YOLO11n. Its mean mAP@0.5:0.95 reached 65.90%, which was 2.29 percentage points higher than the 63.61% achieved by YOLO11n. The standard deviations of all metrics for both models remained within relatively small ranges, indicating that the results of the independent training runs were generally concentrated.

Taken together, ESD-YOLO consistently maintained an advantage in mAP@0.5:0.95 across different random seeds, and its mean performance over multiple independent training runs remained higher than that of YOLO11n. These results indicate that the improvement in this metric exhibits good stability and reproducibility.

#### 3.4.3. Comparison of Performance Metrics by Category

To further evaluate the performance of ESD-YOLO in detecting different termite species, mAP@0.5 and mAP@0.5:0.95 were calculated for each model on the five termite classes: CF, MB, OF, RF, and RC. The results are shown in Table 8.

As shown in Table 8, ESD-YOLO achieved mAP@0.5 values of 97.74%, 98.48%, 96.51%, 98.12%, and 98.41% for the five termite classes, respectively, all of which remained at a high level. This indicates that the model can effectively detect termite targets across different classes. Compared with the baseline YOLO11n, ESD-YOLO achieved higher mAP@0.5:0.95 values for all five termite species. Specifically, mAP@0.5:0.95 increased from 66.64% to 68.29% for CF, from 61.18% to 62.16% for MB, from 60.71% to 62.34% for OF, from 65.01% to 67.22% for RF, and from 66.21% to 69.57% for RC. These results indicate that ESD-YOLO improves bounding-box localization accuracy across different termite classes.

From the class-wise results, ESD-YOLO achieved mAP@0.5:0.95 values of 62.16% and 62.34% for MB and OF, respectively, which were lower than those for the other three termite classes. This indicates that these two classes are more difficult to localize precisely under high IoU thresholds. This may be related to similarities in termite body color, morphological boundaries, and background texture. Under complex backgrounds such as wood chips, soil, and termite galleries, the model is more susceptible to interference when identifying target edges. Nevertheless, the mAP@0.5:0.95 values of ESD-YOLO for these two termite classes were still higher than those of the other comparison models, indicating that the proposed model maintains good localization capability for more challenging classes.

#### 3.4.4. Comparison of Performance Across Relative Target Scales

To further analyze the detection performance of ESD-YOLO for termite targets at different relative scales, a relative-scale stratification experiment was conducted based on the distribution of ground-truth bounding-box areas in the dataset. Because the scale distribution of termite targets was relatively concentrated, fixed scale thresholds commonly used in general-purpose datasets were not adopted. Instead, the relative-scale groups were defined according to the bounding-box area distribution of all 7421 valid termite instances. Using the first quartile (Q1 = 1144.00 pixels^2^) and the third quartile (Q3 = 1739.98 pixels^2^) as thresholds, targets with a bounding-box area of A ≤ 1144.00 pixels^2^ were defined as relatively small targets, those with 1144.00 < A ≤ 1739.98 pixels^2^ as medium-scale targets, and those with A > 1739.98 pixels^2^ as relatively large targets. Here, A denotes the ground-truth bounding-box area in images with a resolution of 640 × 640 pixels. The numbers of targets in each relative-scale group are shown in Table 9.

Based on the above scale stratification, the best-performing YOLO11n and ESD-YOLO weights obtained from the original experiments were evaluated on the same test set using identical inference settings. AP@0.5 and AP@0.5:0.95 were calculated separately for the three relative-scale groups. This experiment did not alter the model training process; the test instances were stratified only at the evaluation stage according to their ground-truth bounding-box areas to compare the detection performance of the two models across different relative target scales. The results are presented in Table 10.

As shown in Table 10, ESD-YOLO achieved the most pronounced performance improvement for relatively small targets. Compared with YOLO11n, its AP@0.5 increased from 89.88% to 91.73%, while AP@0.5:0.95 increased from 51.83% to 53.70%, corresponding to improvements of 1.85 and 1.87 percentage points, respectively. For medium-scale targets, AP@0.5 and AP@0.5:0.95 increased by 1.10 and 1.34 percentage points, respectively. For relatively large targets, AP@0.5 decreased slightly from 99.18% to 98.60%, whereas AP@0.5:0.95 increased from 65.27% to 66.71%, an improvement of 1.44 percentage points. These results show that the performance changes in ESD-YOLO were not fully consistent across different target scales. Nevertheless, relatively small targets achieved the largest gains in both evaluation metrics among the three scale groups, suggesting that the proposed modifications are particularly beneficial for termite targets occupying smaller spatial regions in the image. Meanwhile, the slight decrease in AP@0.5 for relatively large targets indicates that the improvements are not uniform across all target scales and evaluation metrics. Therefore, the main advantage of ESD-YOLO is reflected in the detection of relatively small targets and improved localization performance under stricter IoU thresholds.

### 3.5. Visualization of Detection Results for Each Model

**Figure 6:**
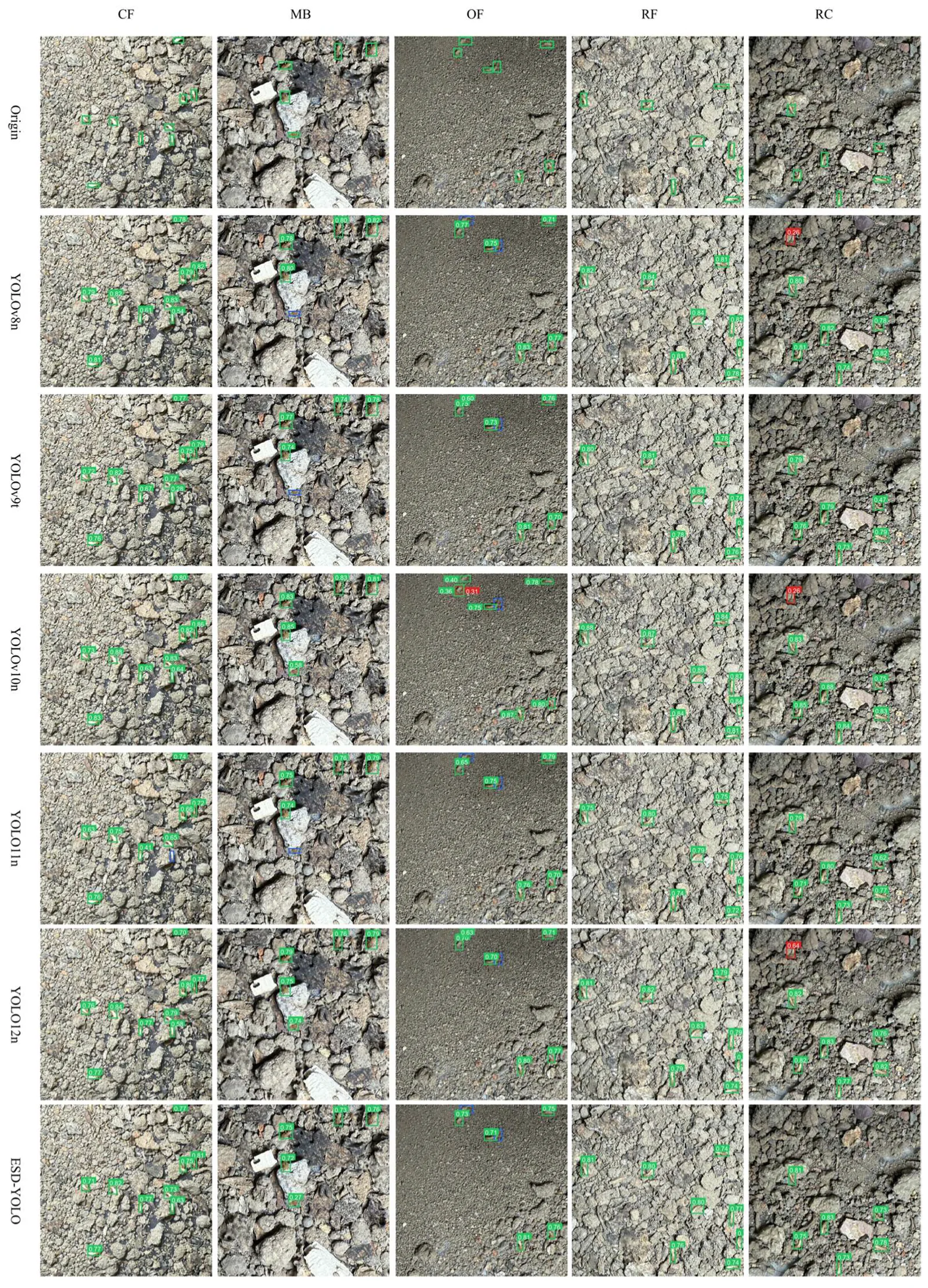
Visual detection results of different models on samples of five termite species. Green boxes indicate correct detections, blue boxes indicate missed targets, and red boxes indicate false positives.

To visually compare the detection performance of different models under complex backgrounds, representative images containing multiple targets were selected from the five termite classes for visualization analysis. The results are shown in Figure 6. Horizontally, the images correspond to CF, MB, OF, RF, and RC, respectively. Vertically, they show the original images and the detection results of YOLOv8n, YOLOv9t, YOLOv10n, YOLO11n, YOLO12n, and ESD-YOLO. Green boxes indicate correct detections, blue boxes indicate missed targets, and red boxes indicate false positives. The detection statistics of each model on these representative samples are shown in Table 11.

As shown in Figure 6, all models detected most termite targets, but differences in false positives and missed detections were still observed in regions with dense targets, small targets, or unclear boundaries. YOLO11n produced no false positives in these representative samples but missed relatively more targets, whereas YOLOv8n produced both missed detections and false positives. YOLOv9t showed relatively stable detection results, although differences in bounding-box coverage were still visible for some targets. YOLOv10n and YOLO12n each missed only one target, indicating high Recall, but produced two and one false positives, respectively. In contrast, ESD-YOLO produced no false positives in these representative samples and missed only two targets, while maintaining relatively consistent bounding-box coverage of the target regions.

Table 11 shows that the representative samples in Figure 6 contained 35 true termite targets. ESD-YOLO achieved Precision and Recall values of 100.00% and 94.29%, respectively, with an average confidence score of 0.745. Compared with the baseline YOLO11n, ESD-YOLO reduced the number of missed detections from 4 to 2 while maintaining zero false positives, and improved Recall from 88.57% to 94.29%. Compared with YOLOv10n and YOLO12n, ESD-YOLO had slightly more missed detections but produced no false positives, indicating better false-positive control.

Based on the detection results for different classes, ESD-YOLO produced no false positives or missed detections in the CF, MB, RF, and RC samples, whereas missed detections were mainly concentrated in the OF sample. This may be related to the small size of OF individuals, the low contrast between their bodies and the surrounding environment, and occlusion among adjacent individuals. Figure 6 and Table 11 show, at the representative-sample level, that ESD-YOLO can effectively control false positives while maintaining a high detection rate under complex backgrounds and achieving relatively stable detection-box coverage.

### 3.6. Multi-Layer Feature Response Analysis Based on Grad-CAM

To analyze the feature responses of ESD-YOLO at different network levels, Gradient-weighted Class Activation Mapping (Grad-CAM) was used for visualization. Grad-CAM obtains the contribution weights of different channels by calculating the gradients of a target score with respect to the feature maps and projects the weighted feature responses onto the input image space. Grad-CAM has increasingly been applied to the interpretability analysis of object detectors under complex natural backgrounds, where saliency responses at different network levels can help reveal the hierarchical feature representations learned during feature extraction [41].

In this study, the joint class score of valid termite detections in an image was used as the backpropagation target for Grad-CAM. Specifically, the predicted class scores of all valid detected instances were summed as follows:

(14) $$y=\sum_{i=1}^{N}s_{i}$$

**Figure 7:**
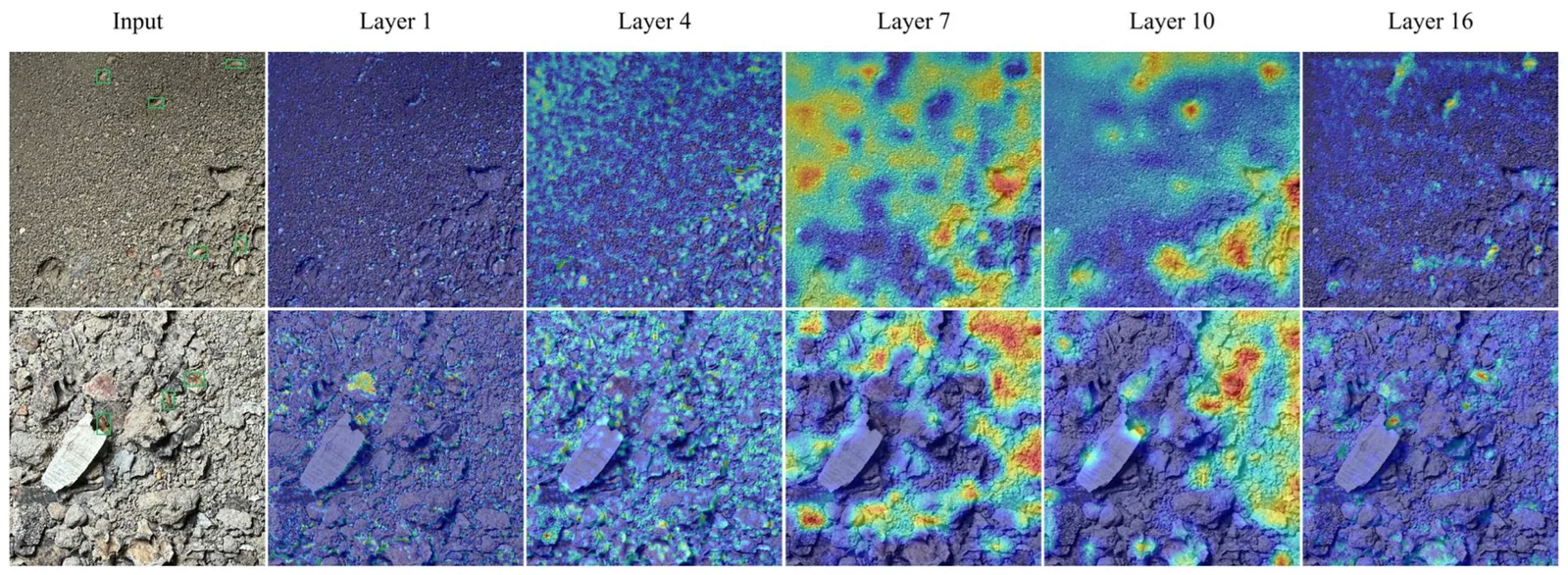
Grad-CAM visualization of feature responses at different network layers of ESD-YOLO. Green boxes indicate ground-truth bounding boxes. Warm colors indicate relatively high feature responses, whereas cool colors indicate relatively low feature responses.

In Equation (14), N denotes the number of valid termite detections included in the analysis for the current image, and $s_{i}$ denotes the predicted class score corresponding to the i-th detected instance. Layer 1, Layer 4, Layer 7, Layer 10, and Layer 16 of ESD-YOLO were selected for visualization. The same Grad-CAM computation and visualization settings were applied to all selected layers, and the results are shown in Figure 7. Warm colors indicate relatively high feature responses, whereas cool colors indicate relatively low feature responses.

As shown in Figure 7, ESD-YOLO exhibited clearly different spatial response patterns across network levels. At the shallow feature stage, the responses of Layer 1 were relatively fragmented and were mainly distributed along termite body edges, soil particles, and other regions with local texture variations. Layer 4 retained rich local edge and texture information while also showing noticeable responses to some termite bodies and their surrounding regions. As feature extraction progressed, the responses of the intermediate layers, Layer 7 and Layer 10, gradually expanded from dispersed local regions to larger continuous areas, exhibiting more evident spatial contextual characteristics. At this stage, the high-response regions were not confined to the termite targets themselves; some background regions containing soil, debris, and pronounced structural variations also showed responses, indicating that the model incorporated spatial information from both the targets and their surroundings.

At the deep feature stage, the responses of Layer 16 became more localized and discrete again. Noticeable responses were observed around multiple termite targets and their neighboring regions, whereas large continuous activation areas were reduced. Similar hierarchical response patterns were observed in both representative samples, reflecting a progressive transition in ESD-YOLO from shallow local texture and edge representations, through intermediate spatial context integration, to deeper target-related feature responses. These visualizations reflect differences in prediction-related feature responses across network levels; however, Grad-CAM itself does not provide direct causal evidence that any specific module suppresses background interference or is responsible for the observed performance improvements.

## 4. Discussion

ESD-YOLO achieved favorable overall performance in the multi-class termite detection task under complex backgrounds, although the evaluation metrics did not change in a fully consistent manner. Compared with the baseline YOLO11n, the Precision of ESD-YOLO increased from 93.32% to 95.39%, an improvement of 2.07 percentage points, whereas Recall decreased slightly from 97.01% to 96.33%, a reduction of 0.68 percentage points. This indicates a trade-off between reducing false-positive predictions and maintaining target detection capability. The improvement in Precision suggests that ESD-YOLO can more effectively reduce false detections caused by complex background textures, whereas the slight decrease in Recall indicates that some challenging targets with low contrast, occlusion, or blurred boundaries may still be missed. Meanwhile, ESD-YOLO improved mAP@0.5 and mAP@0.5:0.95 by 0.35 and 1.97 percentage points, respectively, compared with YOLO11n. The results of multiple independent training runs further showed that the mean mAP@0.5:0.95 of ESD-YOLO was 65.90%, higher than the 63.61% of YOLO11n, and this advantage was consistently observed across all five random seeds. Because the baseline model had already achieved a high mAP@0.5 of 97.50%, the remaining room for improvement in basic target detection was relatively limited. In contrast, mAP@0.5:0.95 evaluates performance across multiple stricter IoU thresholds and places higher requirements on the overlap between predicted and ground-truth bounding boxes. Therefore, the larger improvement in this metric suggests that the performance gains of ESD-YOLO are mainly reflected in false-positive control and bounding-box localization accuracy rather than in simply increasing the target detection rate. For termite monitoring under complex backgrounds, this Precision–Recall trade-off has practical relevance. Background textures such as wood chips, soil particles, stones, and termite-gallery edges can generate local visual patterns similar to termite bodies, and excessive false-positive predictions may increase errors in subsequent manual verification, individual counting, and activity-area analysis. Therefore, improving Precision and localization accuracy while only slightly reducing Recall may help enhance the reliability of the detection results. However, the decrease in Recall also indicates a remaining risk of missed detections for some challenging targets. In future practical applications, the detection confidence threshold can be adjusted according to the tolerance for false positives and false negatives in different monitoring tasks to obtain a Precision–Recall operating point that is better suited to specific application requirements.

From the perspective of specific application tasks, existing deep learning studies on termites have mainly focused on swarming-termite monitoring and single-class termite detection. Wang et al. proposed YOLOv11-ST for the detection and counting of swarming termites, achieving a Recall of 87.32% and an mAP@0.5 of 93.21%, with an average counting accuracy of 91.2% using the associated counting method [19]. This approach primarily monitors swarming activity to reflect termite dispersal and population growth dynamics [42], thereby providing a basis for early warning of termite infestations in agricultural and forestry systems. In contrast, ESD-YOLO is designed for the direct detection and localization of non-swarming termite individuals, with an emphasis on obtaining information on the class, number, and spatial location of active termites, which can support on-site verification of termite activity areas and targeted control. Zou et al. proposed YOLO-HERA, an improved YOLOv8s-based model for real-time detection of a single termite class in complex environments, achieving a 4.1% improvement in AP@0.5 and a 3% increase in accuracy over the original YOLOv8s [20]. Unlike that study, which focused on single-class termite detection, ESD-YOLO extends detection and localization to five termite classes. While maintaining relatively low parameter counts and computational cost, the model achieves high detection accuracy and can provide richer fundamental information for multi-class identification and localization in practical intelligent termite monitoring.

This study still has several limitations. First, the data were collected mainly under controlled conditions. Although complex background elements such as wood chips, soil, stones, and termite galleries were introduced, real-world field monitoring environments are more variable. Factors such as uneven illumination, shadow occlusion, background clutter, target overlap, and image blur may increase the risk of false positives and false negatives, and the robustness of the model under real complex environments therefore requires further validation. Second, the sample sources and acquisition scenarios in the current dataset remain relatively limited. Although the training, validation, and test sets were separated at the acquisition-session and physical rearing-box levels, they were not independently partitioned at the colony or individual-termite levels. Therefore, complete independence among the data subsets could not be guaranteed at these two levels. Future work should adopt stricter colony- and individual-level dataset partitioning to provide a more rigorous evaluation of model generalizability. In addition, the model has not yet been validated through deployment in real monitoring scenarios. Practical applications will require further assessment of the effects of terminal-device computational capacity, inference speed, and variations in image quality on detection performance. Finally, both model training and evaluation in this study were based exclusively on termite images, and the generalization capability of the model to other insect species has not yet been verified. Different insects may differ substantially in body size, coloration, morphology, and activity background, potentially leading to shifts in feature distributions that may limit direct transfer of the model to new insect categories without further adaptation. Future work should therefore expand the termite image dataset by incorporating more real-world field monitoring samples and evaluate model generalization and engineering applicability through cross-scenario testing, independent external test sets, and edge-device deployment experiments. Cross-species detection and adaptation studies involving different insect species should also be further investigated.

## 5. Conclusions

This study addressed the multi-class termite detection task under controlled complex-background conditions and constructed an image dataset containing five termite species, *Coptotermes formosanus*, *Macrotermes barneyi*, *Odontotermes formosanus*, *Reticulitermes flaviceps*, and *Reticulitermes chinensis*, comprising 3160 images and 7421 valid termite instances. To address the challenges posed by the small target size, strong background interference, subtle inter-class differences, and indistinct boundaries of termites, an improved YOLO11n-based model, ESD-YOLO, was developed. Experimental results showed that ESD-YOLO achieved Precision, Recall, mAP@0.5, and mAP@0.5:0.95 values of 95.39%, 96.33%, 97.85%, and 65.92%, respectively, with 2.67 M parameters and 6.68 G FLOPs, demonstrating favorable detection performance while maintaining relatively low model complexity. These results validate the effectiveness of ESD-YOLO on the controlled complex-background dataset constructed in this study and provide a methodological reference for automated termite detection under complex-background conditions.

## Figures and Tables

**Table 1:** Composition and split of the termite image dataset.

Class	Train	Val	Test	Total	Instances	Avg. Inst./Image
CF	349	50	115	514	1352	2.63
MB	429	61	110	600	1462	2.44
OF	419	63	118	600	1294	2.16
RF	515	69	131	715	2041	2.85
RC	500	73	158	731	1272	1.74
Total	2212	316	632	3160	7421	2.35

Note: Train, Val, and Test denote the numbers of training, validation, and test images, respectively. Instances denotes the total number of termite instances, and Avg. inst./image denotes the average number of termite instances per image.

**Table 2:** Statistics of bounding-box areas for termite instances.

Statistic	Bounding-Box Area (Pixels^2^)	Image Area Ratio (%)
Mean	1456.95	0.36
Median	1410.01	0.34
Q1 (25th percentile)	1144.00	0.28
Q3 (75th percentile)	1739.98	0.42
Min–Max	406.00–3653.98	0.10–0.89

Note: Image area ratio denotes the proportion of the total image area occupied by an individual bounding box. All statistics were calculated from images with a resolution of 640 × 640 pixels.

**Table 3:** Main training parameter settings.

Parameter	Value
Image size	640
Epochs	300
Batch size	48
Optimizer	SGD
Initial learning rate	0.01
Final learning rate factor	0.01
Momentum	0.937
Weight decay	0.0005
Automatic mixed precision	False
Seed	0

**Table 4:** Ablation results of different improved modules.

Index	C3k2-EMA	DySample	SPPF-DGP	Precision/%	Recall/%	mAP@0.5/%	mAP@0.5:0.95/%	Params/M	FLOPs/G
1	×	×	×	93.32	97.01	97.50	63.95	2.36	6.44
2	×	×	√	94.87	96.46	97.40	64.48	2.36	6.51
3	×	√	×	94.52	96.74	97.60	64.99	2.37	6.57
4	√	×	×	95.57	96.84	97.96	65.51	2.37	6.50
5	√	√	×	93.55	97.48	97.72	65.02	2.42	6.61
6	×	√	√	95.16	96.75	97.89	65.46	2.42	6.65
7	√	×	√	94.68	97.31	97.97	65.64	2.43	6.59
8	√	√	√	95.39	96.33	97.85	65.92	2.67	6.68

Note: “√” and “×” indicate the inclusion and exclusion of the corresponding module, respectively.

**Table 5:** Overall performance comparison of different object detection models.

Model	Precision/%	Recall/%	mAP@0.5/%	mAP@0.5:0.95/%	Params/M	FLOPs/G	FPS
Faster R-CNN	74.31	76.67	85.98	49.44	41.37	269.02	20.08
RetinaNet-ResNet50	88.98	91.87	95.00	56.89	32.31	257.18	19.56
RT-DETR-R18	90.89	93.47	95.20	57.59	16.98	53.47	30.35
YOLOv8n	94.55	95.54	97.63	62.69	2.69	6.95	48.89
YOLOv9t	94.51	96.08	97.68	63.69	1.77	6.70	23.58
YOLOv10n	93.58	96.19	97.34	62.37	2.71	8.40	45.63
YOLO11n	93.32	97.01	97.50	63.95	2.36	6.44	40.99
YOLO12n	93.98	96.52	97.13	61.09	2.52	5.98	29.99
ESD-YOLO	95.39	96.33	97.85	65.92	2.67	6.68	29.81

**Table 6:** Detection performance of YOLO11n and ESD-YOLO under different random seeds.

Model	Seed	Precision (%)	Recall (%)	mAP@0.5 (%)	mAP@0.5:0.95 (%)
YOLO11n	0	93.32	97.01	97.50	63.95
	1	94.63	96.95	97.88	63.43
	2	93.87	97.37	97.70	63.53
	3	95.28	96.64	97.73	63.88
	4	94.30	96.71	97.68	63.28
ESD-YOLO	0	95.39	96.33	97.85	65.92
	1	95.59	96.94	98.18	66.49
	2	94.36	96.89	97.61	65.79
	3	94.04	97.63	97.66	65.18
	4	94.07	97.48	97.93	66.13

**Table 7:** Statistical comparison of independent training results for YOLO11n and ESD-YOLO.

Model	Precision (%)		Recall (%)		mAP@0.5 (%)		mAP@0.5:0.95 (%)	
	Mean	SD	Mean	SD	Mean	SD	Mean	SD
YOLO11n	94.28	0.74	96.94	0.29	97.70	0.14	63.61	0.29
ESD-YOLO	94.69	0.74	97.05	0.52	97.85	0.23	65.90	0.48

Note: Mean denotes the average of five independent training runs, and SD denotes the sample standard deviation.

**Table 8:** Detection performance comparison of different models for each termite class.

	Classes									
Model	CF		MB		OF		RF		RC	
	I	II	I	II	I	II	I	II	I	II
Faster R-CNN	78.62	47.73	93.37	52.74	76.87	40.47	94.28	55.21	86.75	51.06
RetinaNet-ResNet50	94.71	56.92	97.78	56.76	89.42	49.47	96.40	58.97	96.69	62.31
RT-DETR-R18	93.73	58.81	96.52	54.53	90.84	51.84	97.23	59.50	97.68	63.26
YOLOv8n	96.64	63.71	98.12	59.52	96.46	57.94	98.01	64.83	98.91	67.43
YOLOv9t	96.28	64.89	98.51	60.43	96.75	58.87	98.16	65.38	98.70	68.86
YOLOv10n	96.09	64.40	98.46	60.32	95.60	57.19	97.88	64.18	98.66	65.78
YOLO11n	96.60	66.64	98.35	61.18	96.05	60.71	97.91	65.01	98.58	66.21
YOLO12n	95.85	61.60	98.32	58.87	94.63	55.53	98.54	63.31	98.31	66.16
ESD-YOLO	97.74	68.29	98.48	62.16	96.51	62.34	98.12	67.22	98.41	69.57

Note: I denotes mAP@0.5, and II denotes mAP@0.5:0.95.

**Table 9:** Relative target-scale stratification and instance distribution.

Relative Scale	Area Range, A (Pixels^2^)	All Instances	Test Instances
Relatively small	≤1144.00	1889	426
Medium	1144.00–1739.98	3681	762
Relatively large	>1739.98	1851	366
Total	—	7421	1554

**Table 10:** Detection performance of YOLO11n and ESD-YOLO at different relative target scales.

Model	Relatively Small		Medium		Relatively Large	
	I	II	I	II	I	II
YOLO11n	89.88	51.83	96.28	62.69	99.18	65.27
ESD-YOLO	91.73	53.70	97.38	64.03	98.60	66.71
Improvement	+1.85	+1.87	+1.10	+1.34	−0.58	+1.44

Note: I denotes AP@0.5, and II denotes AP@0.5:0.95.

**Table 11:** Detection statistics of different models on the representative samples shown in Figure 6.

Model	True Targets	Predicted Boxes	TP	FP	FN	Precision/%	Recall/%	Avg. Confidence
YOLOv8n	35	33	32	1	3	96.97	91.43	0.768
YOLOv9t	35	33	33	0	2	100.00	94.29	0.731
YOLOv10n	35	36	34	2	1	94.44	97.14	0.748
YOLO11n	35	31	31	0	4	100.00	88.57	0.726
YOLO12n	35	35	34	1	1	97.14	97.14	0.743
ESD-YOLO	35	33	33	0	2	100.00	94.29	0.745

## Data Availability

The source code and sample data used for the current study are available at https://github.com/weiling-luhz/ESD-YOLO-Termite-Detection.git (accessed on 18 August 2026).
